# Misinformation About and Interest in Chlorine Dioxide During the COVID-19 Pandemic in Mexico Identified Using Google Trends Data: Infodemiology Study

**DOI:** 10.2196/29894

**Published:** 2022-01-27

**Authors:** Jonathan Matias Chejfec-Ciociano, Juan Pablo Martínez-Herrera, Alexa Darianna Parra-Guerra, Ricardo Chejfec, Francisco José Barbosa-Camacho, Juan Carlos Ibarrola-Peña, Gabino Cervantes-Guevara, Guillermo Alonso Cervantes-Cardona, Clotilde Fuentes-Orozco, Enrique Cervantes-Pérez, Benjamín García-Reyna, Alejandro González-Ojeda

**Affiliations:** 1 Unidad de Investigación Biomédica 02 Hospital de Especialidades del Centro Médico Nacional de Occidente Instituto Mexicano del Seguro Social Guadalajara Mexico; 2 Max Bell School of Public Policy McGill University Montreal, QC Canada; 3 Hospital Civil de Guadalajara “Fray Antonio Alcalde” Universidad de Guadalajara Guadalajara Mexico; 4 Departamento de Bienestar y Desarrollo Sustentable Centro Universitario del Norte Universidad de Guadalajara Colotlán Mexico; 5 Departamento de Disciplinas Filosófico, Metodológicas e Instrumentales Centro Universitario de Ciencias de la Salud Universidad de Guadalajara Guadalajara Mexico; 6 Departamento de Nutriología Clínica Instituto Nacional de Ciencias Médicas y Nutrición “Salvador Zubirán” Ciudad de Mexico Mexico; 7 Centro Universitario del Norte Universidad de Guadalajara Colotlan Mexico

**Keywords:** coronavirus, COVID-19, Google Trends, chlorine dioxide, COVID-19 misinformation, public health surveillance, infodemiology, internet behavior, digital epidemiology, internet, mHealth, mobile health, pandemic, tele-epidemiology

## Abstract

**Background:**

The COVID-19 pandemic has prompted the increasing popularity of several emerging therapies or preventives that lack scientific evidence or go against medical directives. One such therapy involves the consumption of chlorine dioxide, which is commonly used in the cleaning industry and is available commercially as a mineral solution. This substance has been promoted as a preventive or treatment agent for several diseases, including SARS-CoV-2 infection. As interest in chlorine dioxide has grown since the start of the pandemic, health agencies, institutions, and organizations worldwide have tried to discourage and restrict the consumption of this substance.

**Objective:**

The aim of this study is to analyze search engine trends in Mexico to evaluate changes in public interest in chlorine dioxide since the beginning of the COVID-19 pandemic.

**Methods:**

We retrieved public query data for the Spanish equivalent of the term “chlorine dioxide” from the Google Trends platform. The location was set to Mexico, and the time frame was from March 3, 2019, to February 21, 2021. A descriptive analysis was performed. The Kruskal-Wallis and Dunn tests were used to identify significant changes in search volumes for this term between four consecutive time periods, each of 13 weeks, from March 1, 2020, to February 27, 2021.

**Results:**

From the start of the pandemic in Mexico (February 2020), an upward trend was observed in the number of searches compared with that in 2019. Maximum volume trends were recorded during the week of July 19-25, 2020. The search volumes declined between September and November 2020, but another peak was registered in December 2020 through February 2021, which reached a maximum value on January 10. Percentage change from the first to the fourth time periods was +312.85, –71.35, and +228.18, respectively. Pairwise comparisons using the Kruskal-Wallis and Dunn tests showed significant differences between the four periods (*P*<.001).

**Conclusions:**

Misinformation is a public health risk because it can lower compliance with the recommended measures and encourage the use of therapies that have not been proven safe. The ingestion of chlorine dioxide presents a danger to the population, and several adverse reactions have been reported. Programs should be implemented to direct those interested in this substance to accurate medical information.

## Introduction

### COVID-19 and Therapies

In December 2019, a new strain of coronavirus was detected for the first time in the city of Wuhan, China. The causative agent was identified as SARS-CoV-2. This virus spread across 218 countries and caused a global health crisis [1,2]. In March 2020, the disease was declared a pandemic by the World Health Organization [2]. In Mexico, the first case of COVID-19 was detected on February 27, 2020, in Mexico City. On March 30, 2020, with 328 confirmed cases and 12 deaths, a national health emergency was declared, given the exponential increase in confirmed cases and deaths from the disease [3]. According to the Pan American Health Organization on November 21, 2021, a total of 3,867,976 cases and 292,850 deaths from COVID-19 have been confirmed in Mexico, with a cumulative incidence rate of 2999.1 per 100,000 people [4].

Given the lack of specific preventive measures or treatment for COVID-19 during the first global outbreak (February 2020), several alleged therapies and preventive measures have emerged, although most have not been scientifically proven. Because crises such as pandemics usually generate a variety of psychological reactions, it is likely emotions such as fear drive the population to seek alternatives to protect themselves [5]. Misinformation about diseases has been well documented in the literature, usually revolving around causation, transmission, and potential cures of predominantly infectious diseases. This phenomenon has been reported in the past for conditions such as leprosy, tuberculosis, and influenza, among others [6]. Belief in such misinformation can be dangerous because it may reduce the adoption of proven health and hygiene measures, and create a false sense of security. These products may also pose other health risks given the lack of evidence of their safety. Bogus therapies tend to be widely promoted over a short time, and people can be predisposed to following them without questioning their authenticity or whether there is supporting evidence [6]. Some bogus therapies popular during the COVID-19 pandemic include eating garlic, turmeric, and lemon under the assumption that these substances have antimicrobial properties [6].

### Chlorine Dioxide and Misinformation

Chlorine dioxide is a chemical compound commonly used as a bleach and disinfectant in industrial processes and water purification treatment [7,8]. Nevertheless, sellers and distributors have claimed that this substance may serve as treatment for multiple pathologies such as autism, Ebola virus, cancer, hepatitis, diabetes, HIV/AIDS, COVID-19, and even depression [9,10]. The commercialized product containing chlorine dioxide or sodium chlorite for health-related issues is advertised in English as chlorine dioxide solution or miracle mineral solution, while in Spanish, it is known as “Dióxido de cloro” [11,12]. Chlorine dioxide solution was commercialized and used in various countries across Europe and America before the COVID-19 pandemic. These products are promoted as nutritional supplements to bypass the strict approval processes required by law for medicines or health treatments [13,14]. Throughout the pandemic, the demand for chlorine dioxide has increased alarmingly worldwide. Since January 2020, the US Food and Drug Administration (FDA) has received reports of serious adverse events in patients who have consumed this substance [12]. Adverse reactions included respiratory failure, disturbance of the heart’s electrical activity, hypotension, acute liver failure, acute kidney injury, hemolytic anemia, vomiting, and severe acute diarrhea [7,15].

Consequently, health agencies, institutions, and organizations worldwide tried to discourage consumption of chlorine dioxide solution by refuting the false claims that painted it as a therapeutic and preventive treatment for COVID-19. In Spain, on May 14, 2010, the Ministry of Health ordered chlorine dioxide solution, which was sold on the internet, to be withdrawn from the market [10]. In the United States, on April 8, 2020, the FDA advised consumers not to buy or ingest any chlorine dioxide–based products because of the lack of scientific evidence of their efficacy or safety [12]. On July 6, 2020, an official statement addressed to health personnel was published on the Mexican government’s official website. The document warned medical personnel not to recommend the use of chlorine dioxide. Later, on July 23, 2020, the Mexican regulatory agency “Comisión Federal para la Protección contra Riesgos Sanitarios” (COFEPRIS) released a statement to the Mexican population informing them of the risk of chlorine dioxide solution or miracle mineral solution, emphasizing that its consumption should be stopped immediately, and encouraging the reporting of any adverse reaction related to its use [16]. On August 18, 2020, the Pan American Health Organization published a post on their Facebook page warning about false information about chlorine dioxide solution use [17].

Several reports have been published on the impact of the internet and social media on the population, misinformation about COVID-19, and the quality of information available online. In a study involving a 27-question survey of 1136 students, Chesser et al [18] reported that only 43% had a high literacy level about COVID-19. Most of this sample reported the internet and social media as their primary source for COVID-19 information. Cuan-Baltazar et al [19] evaluated the quality and readability of the first 110 English and Spanish website results for the search term “Wuhan coronavirus” appearing in the Google search engine on February 6, 2020. Webpages were evaluated using different instruments for online health information. Most of the sample was considered to be of low quality in terms of the information provided [19]. Roozenbeek et al [20] studied the susceptibility to misinformation about COVID-19. Misinformation was perceived as being the most reliable in Mexico, compared with the United Kingdom, Ireland, the United States, and Spain. When analyzing the predictors of susceptibility to misinformation, being older was associated with a lower risk in all countries, except for Mexico, where it was significantly higher [20]. In another study undertaken in May 2020, the top 75 viewed videos using the word “coronavirus” and “COVID-19” were analyzed, and 27.5% of videos were found to contain nonfactual information that achieved 62,042,609 views in total [21].

### Infodemiology and Google Trends

Infodemiology is the science that studies the distributions and determinants of information shared in electronic media concerning public health [22]. The rapid increase in internet users and information published worldwide enables data collection in almost real time. According to a statement published in May 2020 by the National Institute of Statistics and Geography, 56.4% of Mexican households have internet access, reporting entertainment, obtaining information, and communicating as the main activities performed [23,24].

Google Trends is a platform provided by Google that allows one to assess the search frequency of a specific term during a certain time period. The platform tracks words from search queries that users enter into the Google search engine and presents them according to a specified time period and geographic location. The search volume results are presented as a relative search volume (RSV) index, wherein each data point is divided by the total number of searches performed in a specified geographical region within a given time range to provide relative comparisons [25].

Google Trends has been used as a tool to provide insights into population behavior [26] and has played a role in several distinct types of studies during the COVID-19 pandemic. It has been used to investigate the interest of specific diseases such as Kawasaki disease [27], to evaluate societal interest in pornography during the crisis [28], to find correlations between chest pain search volume and acute coronary syndromes hospital admissions [29], and to compare public awareness on the COVID-19 pandemic across different countries [30], among others. Additionally, Walker et al [31] reported statistically significant correlations between daily searches related to loss of smell and COVID-19 new cases and deaths [31,32]. We found only two articles using infodemiology to study interest in chlorine dioxide in Mexico. Both studies used an international comparative analysis to evaluate differences in search trends for chlorine dioxide solution among countries, in which Mexico stood out as one of the countries with the highest number of searches [33,34].

The primary goal of our study was to investigate changes in interest trends for chlorine dioxide prior to and during the COVID-19 pandemic in Mexico using internet search volume in a popular search engine as a proxy. In the process, we also aimed to determine the impact of official governmental communications deterring from the substance’s consumption on the trend. It is hypothesized that during the pandemic in Mexico, RSVs for chlorine dioxide increased. Additionally, we believe this trend is fueled by social and media interactions, stems from unreliable sources, and is poorly influenced by government public health statements.

## Methods

### Data Extraction

The database was created using the Google Trends platform provided by Google. Data on the RSVs were extracted at the national level and by state during the entire period selected. The location was set to Mexico, the category to “All categories” and “Health,” and the time period was specified to March 3, 2019, to February 21, 2021. The data values ranged between 0 and 100. The terms analyzed were the Spanish translations of chlorine dioxide: “dioxido de cloro” and “dióxido de cloro.” Both terms were included to capture searches without the written accent. Because there is no official registration of this product in the legal market, the commercial name varies depending on the producer and distributor. However, we use this term, as it is the one used by most health institutions’ communications and news releases.

### Data Analysis

**Figure 1:**
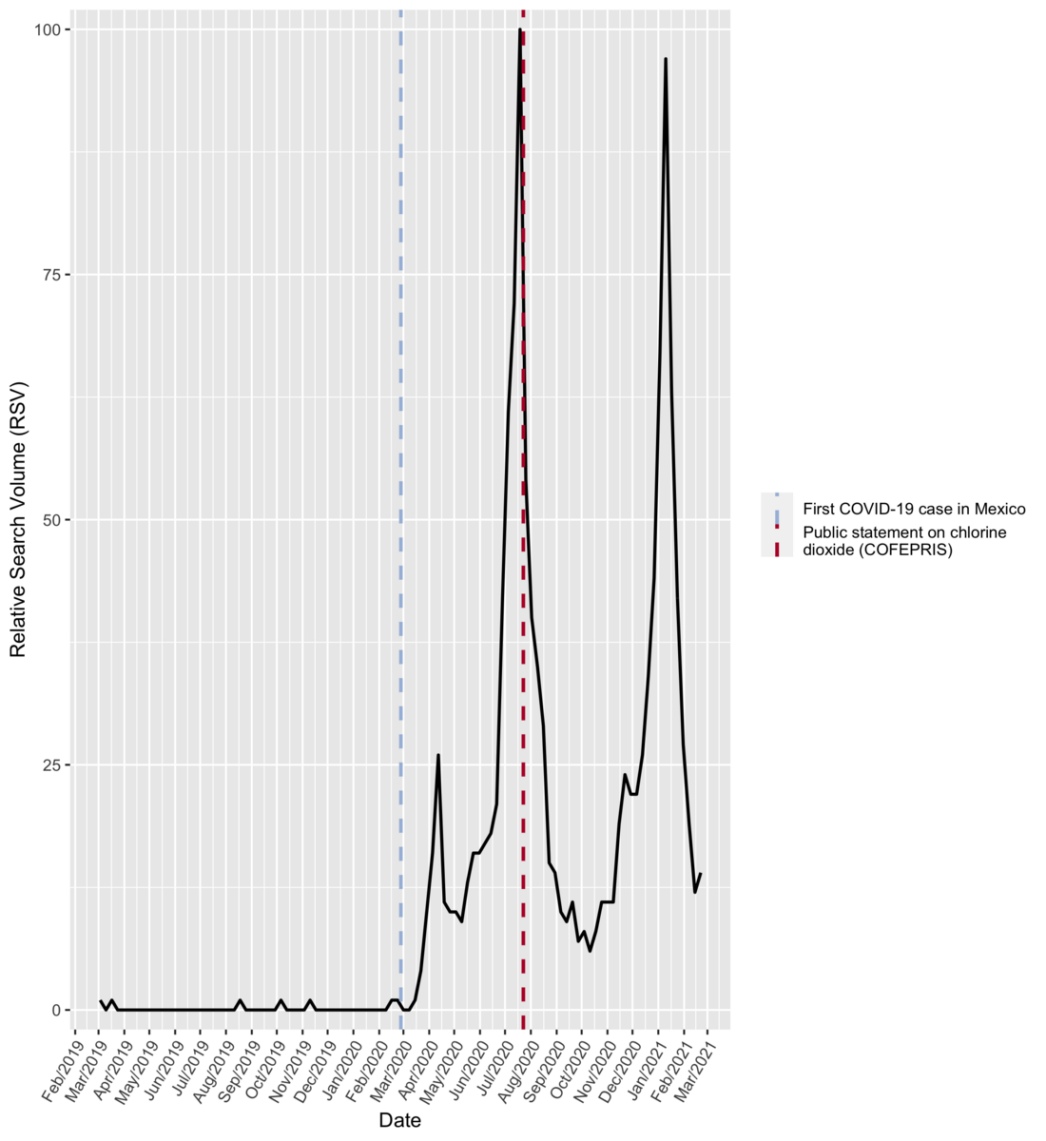
Timeline for the relative search volume index for “chlorine dioxide” in Mexico from March 3, 2019, to February 21, 2021. The first confirmed COVID-19 case in Mexico and governmental public statement about this substance are included in the plot. COFEPRIS: Comisión Federal para la Protección contra Riesgos Sanitarios.

The database was downloaded in CSV format in the time period established according to the number of weekly searches and RSV index. Data were exported to RStudio (version 0.97.551; RStudio, PBC) and SPSS (version 21; IBM Corp) for analysis. Multiple seasonal subseries box plots were analyzed to rule out annual or seasonal patterns. The timeline is presented in Figure 1. An analysis was then performed on data from March 1, 2020, to February 27, 2021.

From March 1, 2020, to February 27, 2021, we recorded data spanning 52 weeks. Four groups of 13 weeks each were created, as it allowed for easy comparisons and enabled us to divide them into equally sized sets. The Kruskal-Wallis test was used to compare the total number of searches for chlorine dioxide in the four time periods since the first case of COVID-19 in Mexico. The post hoc Dunn test was used to identify differences between means.

## Results

**Figure 2:**
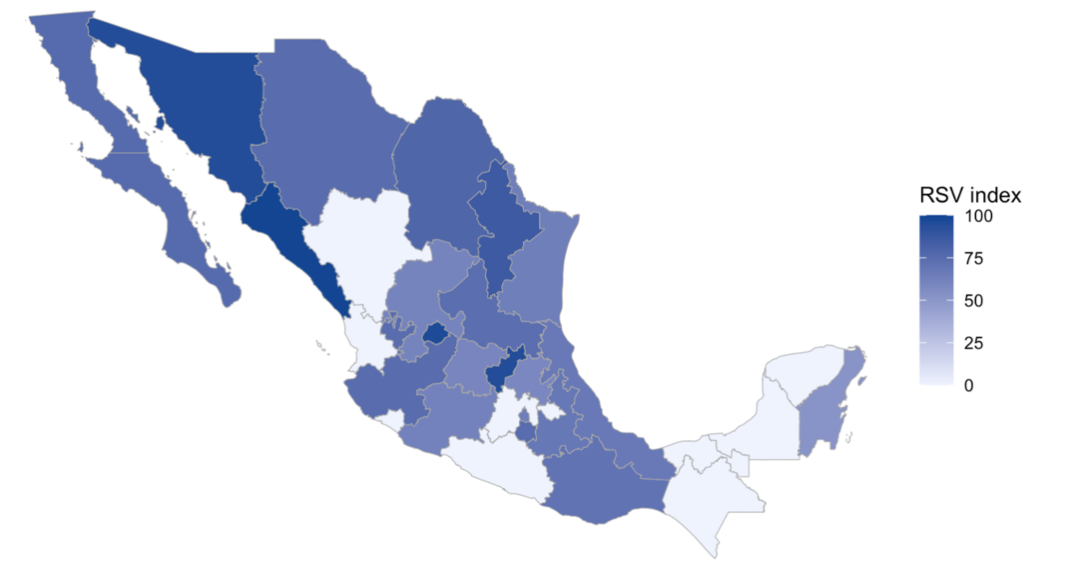
RSV index by Mexican states from March 3, 2019, to February 21, 2021. RSV: relative search volume.

**Figure 3:**
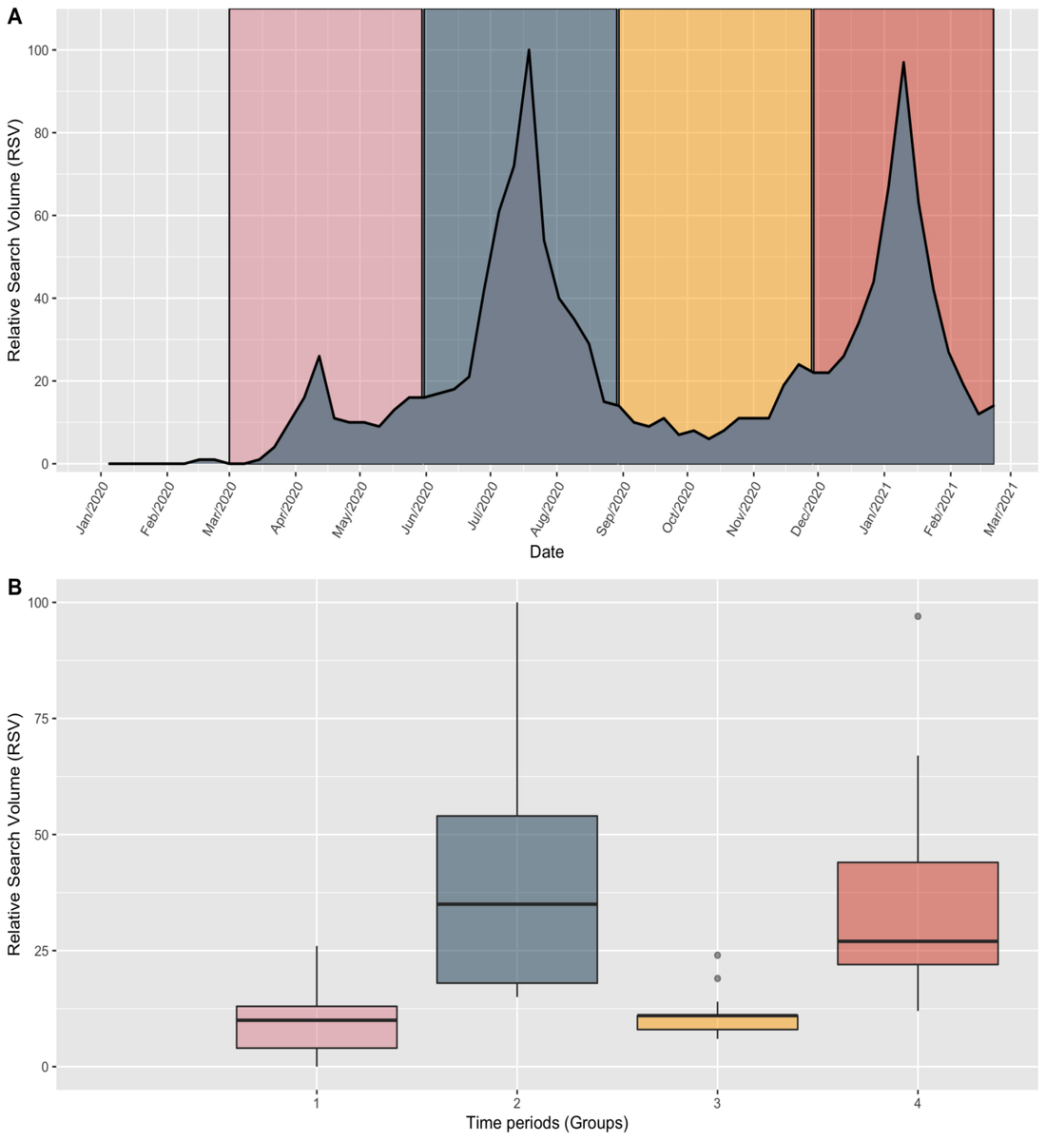
A: Timeline of the RSV index for chlorine dioxide in Mexico since the first confirmed COVID-19 case in Mexico shown according to the different time periods analyzed. B: Data distribution of RSV index values by time period. RSV: relative search volume.

The average RSVs for the term chlorine dioxide in Mexico for the first, second, third, and fourth time periods analyzed were 9.69 (SD 7.38), 40.00 (SD 25.79), 11.46 (SD 5.02), and 37.61 (SD 24.83), respectively. Dates, averages, and SEs are shown in Table 1. Visual analysis of the data since 2019 did not show any seasonal or annual patterns. Since the beginning of the pandemic in Mexico, the specified term’s popularity has increased, as demonstrated by an upward trend in the number of searches. During the first time period (from early March 2020), the number of searches increased slightly, and the search volume was 26 relative to its maximum popularity (set at 100). During the second time period, this term’s highest search volume since 2019 reached 100 in the week of July 19-25, 2020, after which it declined rapidly to 15 on August 23-29. The search volume continued to decline between September and November 2020 during the third period and reached its lowest point (7) on September 27, 2020. At the end of the third period, the volume increased slightly and, during the fourth period, continued in an upward trend that led to a sudden increase to 97 on January 10, 2021. Five weeks later, the volume declined sharply to 12, and the pattern was similar to that in the second time period. A subanalysis was carried out to highlight those states with the greatest search tendency during the aforementioned period. The highest scores were recorded in the following states: Sinaloa (n=100), Aguascalientes (n=96), Querétaro (n=95), Sonora (n=95), and Nuevo León (n=96). No visual geographical relationship was found concerning search trends. The results are presented in Figure 2. The timeline of the search volumes and time periods are shown in Figure 3.

**Table 1:** Time periods analyzed.

Groups	Time period	Weeks	RSV^a^		Percentage change (%)
			Mean (SE)	Minimum to maximum	
Group 1	March 1 to May 30, 2020	13	9.69 (2.04)	0-26	N/A^b^
Group 2	May 31 to August 29, 2020	13	40.00 (7.15)	15-100	312.85
Group 3	August 30 to November 28, 2020	13	11.46 (1.39)	6-24	–71.35
Group 4	November 29, 2020, to February 27, 2021	13	37.61 (6.88)	12-97	228.18

^a^RSV: relative search volume.

^b^N/A: not applicable.

The Kruskal-Wallis test results show a statistically significant difference (*P*<.001) between the mean ranks of at least one pair of groups. Pairwise post hoc comparisons using the Dunn test indicated four significant comparisons. The data distributions are presented in Figure 2. The mean search volume was significantly lower in the first time period (9.69) than in the second (40.0) and fourth (37.61) time periods (*P*<.001 for each comparison). The mean volume was significantly higher in the second (40.0) than in the third (11.46; *P*=.001) time periods. The mean volume was significantly lower in the third than in the fourth (*P*=.001) time period. The differences were not significant between time periods 1 and 3 and between 2 and 4 (*P*>.99 for both).

## Discussion

### Principal Results

Interest in chlorine dioxide grew as the pandemic started in Mexico. We found a significant increase in the number of searches for chlorine dioxide at the beginning of the pandemic in February 2020 in Mexico. Two peaks—in July 2020 and January 2021—are of particular public interest. Differences in statistical significance were demonstrated between the four time periods evaluated, suggesting an unknown mechanism that drives public interest. Maximum interest was recorded during June to August 2020 and December through February 2021, with previous time periods with significantly lower search volumes. Although chlorine dioxide is an essential ingredient of sanitizers used for surfaces and water, the variable of interest likely responded to searches corresponding to the purported product for COVID-19.

The Mexican regulatory agency COFEPRIS published a statement urging citizens to stop the consumption of this substance on July 23, 2020. Sousa-Pinto et al [35] reported media coverage can have a strong influence on RSVs compared with actual epidemic trends or public behavior. Nevertheless, many of these communication channels do not have the processes in place to filter misinformation related to public health issues. Therefore, the dissemination of information by specialized health institutions through these channels is essential in educating the public. Consistent with the hypothesis, this COFEPRIS public statement did not seem to have an effect on the number of searches, as it was released shortly past the first popularity peak and 3 months before the second peak. We believe this may be indicative of the government’s poor ability to disseminate public health information relative to alternative media sources.

Several Latin American artists, singers, and influential people have been reported to promote chlorine dioxide as a protective or therapeutic agent against the disease caused by SARS-CoV-2. Such promotion was carried out through publications on social networks, media interviews, and consumption of chlorine dioxide solution on live television [36-38]. As these communication channels are likely to have had an impact on the population’s interest in chlorine dioxide solution, it is essential to research the primary sources used by the population for health-related advice. Based on the different levels of RSV seen across states, we suspect search trends are likely most influenced by the media that is consumed locally, such as local celebrities, social networks, or television shows.

Ensuring public health statements from COFEPRIS are well disseminated and widely consumed is essential. Facing a public health emergency, it is important to measure the effectiveness of such statements as well as evaluating the best timelines and pathways to relay important messages. As experts identify a gradually growing interest toward potentially adverse treatments, major health institutions ought to prioritize informing about the dangers and redirecting toward reliable sources. In this sense, the lack of regulation seen in many of the channels through which untruthful medical information is often shared, compromises a threat to public health.

On September 20, 2020, two months after the aforementioned COFEPRIS statement was released, more than 100 people protested in Mexico City to demand the use of chlorine dioxide solution in hospitals. This group of people, allegedly led by a group of scientists and doctors, also marched against the use of masks and vaccines [37]. Assuming that this trend is influenced by media that promotes consumption through nonfactual information and does not respond to communications created by public health institutions, we believe that changes in search volumes may be at least partially associated with changes in consumption of chlorine dioxide solution. Currently, there are two case reports published of complications after chlorine dioxide solution prophylactic ingestion for COVID-19 in Mexico, an acute kidney injury and an intestinal perforation [39,40].

### Limitations

Multiple limitations should be considered when interpreting these results. Search volumes were used as a proxy to measure population interest in this product and should not be interpreted as indicative of the number of people consuming chlorine dioxide. Additionally, standardization of data by Google does not allow for comparisons between absolute numbers of search volumes.

The results were also limited to the population with internet access (56.4% of Mexican households) and who used Google as their standard search engine [23,24]. Other media sources such as news coverage and word of mouth may have had a more significant impact on misinformation and consumption of chlorine dioxide solution than the internet.

### Comparison With Prior Work

Misinformation has been associated with negative views about public health measures. In two cross-sectional studies, Bertin et al [41] reported that conspiratorial beliefs negatively predicted participants’ attitudes of and intentions to be vaccinated against COVID-19. This observation also relates to the views of chloroquine. The use of this drug to prevent severe COVID-19 was controversial at first, and after several studies were published, multiple governments and scientific committees disapproved of its use. Being prochloroquine was associated with a negative attitude about COVID-19 vaccination and a preference for alternative over biomedical therapies [41]. Greater susceptibility to misinformation was associated with reduced compliance with public health guidance and a decreased likelihood of being vaccinated or recommending vaccination [42].

Although disinformation is not a new enemy of public health, the internet and social networks can be powerful sources of misinformation among the general population and can contribute to the undermining of public health policies during a pandemic. Myths have a strong cultural influence that drives social impact [6]. Misinformed beliefs are significantly associated with lower levels of digital health literacy, confidence in government, and trust in scientific institutions [43]. Given that the internet and social media have become new tools for seeking health-related information, misinformation must be addressed by the public health realm. Misleading information has been published about the virus and how it spreads; about how to prevent the infection; about who or what is responsible for it; and in attempt to discredit preventive measures, therapies, and vaccines [20,44-46]. The lack of corroboration of the scientific veracity of what is advertised has allowed companies and individuals to profit from deceiving the consumer into buying products lacking medical evidence or government authorization. There is evidence of misinformation about cancer, Ebola and Zika viruses, smoking, and COVID-19 that has been associated with harmful consequences reported on a global scale [46].

Multiple scientific articles have described approaches in which health institutions can respond to misinformation, which we recommend Mexican public health agencies to implement. Strategies such as automatic learning techniques capable of identifying misleading information on the internet; health care institutional use of social media to promote evidence-based information; studies on the dissemination, creation, and consumption of false information on the internet; and the use of infodemiology to analyze trends in population behavior [44-47]. Given that misleading information is distributed among a new generation of social media users, it is time to include infodemiology and internet scientific research in the public health agenda.

### Conclusions

The pandemic stimulated interest in Mexico toward a substance that was previously sold as a prophylaxis or treatment for multiple diseases in other countries without sufficient medical evidence. Multiple potential mechanisms could have been involved in the double peak observed. This interest is likely to influence the level of consumption of this substance; thus, it is necessary to continue investigating its means of dissemination and its impact on the likeliness to believe or propagate more misinformation in Mexico. In addition to collecting data on chlorine dioxide solution consumption, risks, and adverse reactions, future research should study in-depth the effects of misinformation and the role Mexican culture has played in its uptake. We assume that social networks play an essential role in disseminating this data, given the evidence of the medium as a propagator of disinformation. It is crucial to continue analyzing the role these new media platforms play regarding health decisions as well as evaluating the quality of the information available.

## Abbreviations

COFEPRIS: Comisión Federal para la Protección contra Riesgos Sanitarios
FDA: Food and Drug Administration
RSV: relative search volume
